# Use of spirometry among chest physicians and primary care physicians in India

**DOI:** 10.1038/npjpcrm.2016.36

**Published:** 2016-07-07

**Authors:** Nitin Vanjare, Sushmeeta Chhowala, Sapna Madas, Rahul Kodgule, Jaideep Gogtay, Sundeep Salvi

**Affiliations:** 1 Chest Research Foundation, Pune, India; 2 Medical services, Cipla Ltd., Mumbai, India

## Abstract

Although spirometry is the gold-standard diagnostic test for obstructive airways diseases, it remains poorly utilised in clinical practice. We aimed to investigate the use of spirometry across India, the change in its usage over a period of time and to understand the reasons for its under-utilisation. Two nationwide surveys were conducted in the years 2005 and 2013, among four groups of doctors: chest physicians (CPs), general physicians (GenPs), general practitioners (GPs) and paediatricians (Ps). A total of 1,000 physicians from each of the four groups were randomly selected from our database in the years 2005 and 2013. These surveys were conducted in 52 cities and towns across 15 states in India. A questionnaire was administered to the physicians, which captured information about their demographic details, type of practice and use of spirometry. The overall response rates of the physicians in 2005 and 2013 were 42.8% and 54.9%, respectively. Spirometry was reported to be used by 55% CPs, 20% GenPs, 10% GPs and 5% Ps in 2005, and this increased by 30.9% among CPs (*P* value <0.01), 18% among GenPs (*P* value=0.01), 20% among GPs (*P* value: not significant) and 224% among Ps (*P* value <0.01). The reasons for not using spirometry varied between 2005 and 2013. In all, 32.2% of physicians were unaware of which predicted equation they were using. The use of spirometry in India is low, although it seems to have improved over the years. The reasons identified in this study for under-utilisation should be used to address initiatives to improve the use of spirometry in clinical practice.

## Introduction

Spirometry is the gold-standard, guideline-recommended test for the diagnosis of obstructive airways diseases (OADs), including asthma and chronic obstructive pulmonary disease (COPD).^1^ It also helps distinguish between the two diseases, offers a useful index of severity and prognosis and helps guide appropriate pharmacotherapy.^2,3^

Despite these benefits, most physicians in clinical practice rely only on history and clinical examination to make a diagnosis of OAD and to start pharmacotherapy.^4^ This approach leads to both under-diagnosis and over-diagnosis of OAD and sometimes even inappropriate use of pharmacotherapy.^5,6^ In a study of over 20,000 adults from the third National Health and Nutrition Examination Survey III in USA, it was demonstrated that relying on history and clinical examination alone, under-diagnosed OAD by up to 63% compared to use of spirometry.^7^ More worryingly, 44% of those with even severe airways obstruction detected on spirometry were not diagnosed to have OAD by the physicians. Similar observations have been reported from several other countries, including Spain,^8^ Poland,^9^ Canada,^10^ Austria,^11^ China and Japan.^12,13^ These observations highlight the fact that when spirometry is not used a significantly large number of patients with OAD remain undiagnosed.

Unlike measuring blood pressure or obtaining an electrocardiogram, spirometry is an effort-dependent test that requires not only sufficient patient cooperation but also requires a certain amount of training and expertise to perform the test properly and to ensure good quality (ATS/ERS 2005) and sound knowledge to interpret the results.^14^ Moreover, spirometers are not as cheap as blood pressure apparatus or an electrocardiogram machine, although in recent years their cost has come down significantly. Spirometry is not well taught in most medical schools in India, largely because of non-availability of the instrument in medical schools, but also because many teachers are not well versed with spirometry. Although there is a general perception that spirometry is under-utilised in India, there are no data to accept or refute these claims. We therefore undertook this study to investigate the use of spirometry in India and to evaluate whether this has changed with time.

## Results

In the 2005 survey, out of the 1,000 each chest physicians (CPs), general physicians (GenPs), general practitioners (GPs) and paediatricians (Ps) approached, 458, 717, 256 and 209 physicians responded, with an overall response rate of 42.8%, whereas in the 2013 survey 494, 744, 485 and 426 physicians responded, with an overall response rate of 54.9%.

**Figure 1:**
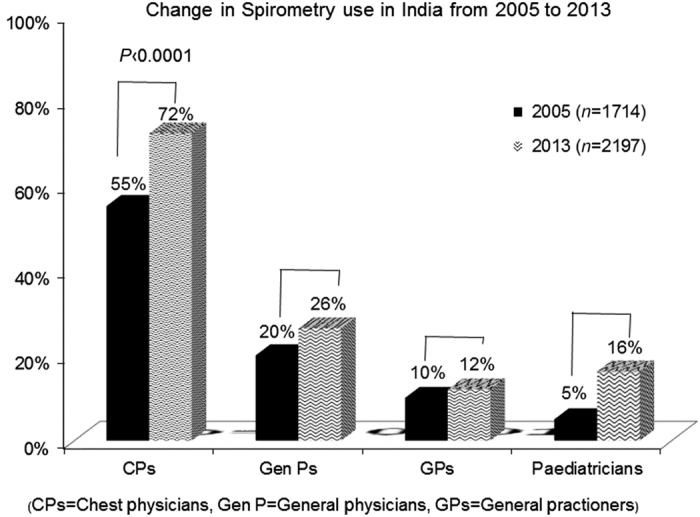
Changes in the use of spirometry by chest physicians (CPs), general physicians (GenPs), general practitioners (GPs) and paediatricians (Ps) between the years 2005 and 2013.

In the 2005 survey, 55% of CPs, 20% of GenPs, 10% of GPs and 5% of Ps reported using spirometry in their practice to diagnose OAD, whereas in the 2013 survey 72% CPs, 26% GenPs, 12% GPs and 16% Ps reported using spirometry in their practice. Therefore, compared with 2005, the use of spirometry in 2013 increased by 30.9% among CPs, 18% among GenPs, 20% among GPs and 224% among Ps (Figure 1).

The reasons for not using spirometry in 2005 as reported by the physicians were as follows: spirometers were expensive (54%), lack of affordability by patients (43%), lack of time by doctors (9%), and difficulty in performing (14%) and interpreting spirometry (19%); in 2013, the respective proportions were 28%, 29%, 32%, 10% and 8%. The detailed reasons for each category of physicians for not using spirometry are given in Table 1.

**Figure 2:**
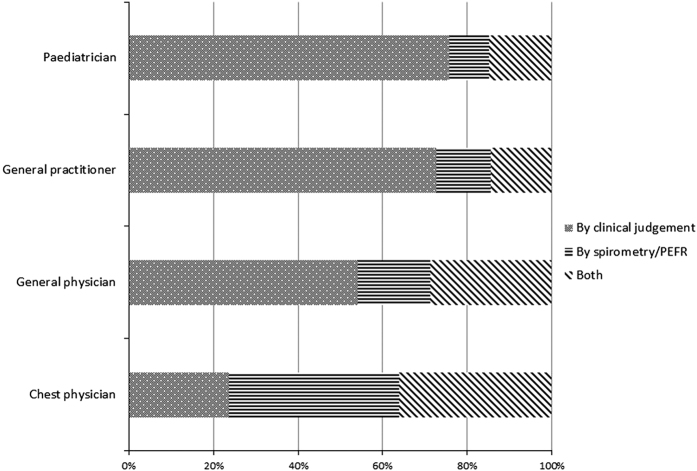
Mode of diagnosis of asthma by chest physicians (CPs), general physicians (GenPs), general practitioners (GPs) and paediatricians (Ps) in the year 2013. In all, 75.72% of Ps, 72.69% of general practitioners, 54.16% of GenPs and 23.57% of CPs diagnosed asthma by clinical judgement; 9.38% of Ps, 12.82% of general practitioners, 17.19% of GenPs and 40.37% of CPs diagnosed asthma by spirometry/peak expiratory flow rate (PEFR); and 14.90% of Ps, 14.50% of general practitioners, 28.65% of GenPs and 36.07% of CPs diagnosed asthma by both clinical judgement and spirometry/PEFR.

**Figure 3:**
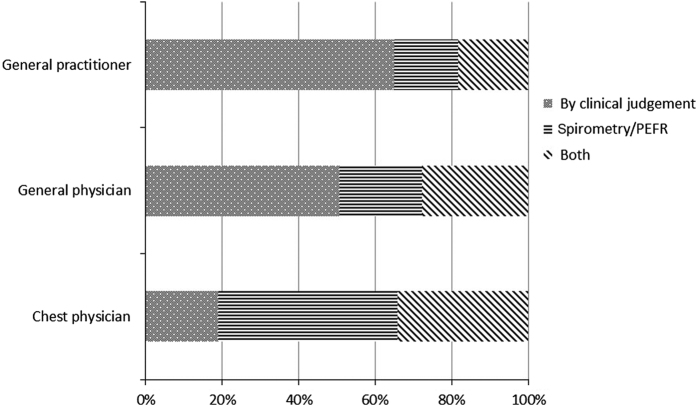
Mode of diagnosis of COPD by chest physicians (CPs), general physicians (GenPs), general practitioners (GPs) and paediatricians (Ps) in the year 2013. In all, 64.90% of general practitioners, 50.55% of GenPs and 18.93% of CPs diagnosed COPD by clinical judgement; 16.78% of general practitioners; 21.70% of GenPs and 46.91% of CPs diagnosed COPD by spirometry/PEFR; and 18.32% general practitioners, 27.75% of GenPs and 34.16% of CPs diagnosed COPD by both clinical judgement and spirometry/ PEFR.

The mode of diagnosis of asthma and COPD by CPs, GenPs, GPs and Ps in clinical practice in 2013 is given in Figures 2 and 3.

There was uncertainty among practitioners about the selection of predicted equations for their population. In all, 32.2% of physicians were unaware of which predicted equation they were using.

## Discussion

### Main findings

We investigated the use of spirometry across India in the year 2005 and repeated it in 2013 to look for changes over time. In 2005, 55% of CPs, 20% of GenPs, 10% of GPs and 5% of Ps reported using spirometry in their practice to diagnose and manage OADs, and these numbers increased by 30.9%, 18%, 20% and 224%, respectively, in the year 2013. Although the use of spirometry has increased in India over time among CPs and primary care physicians, it is still far from what is desired. The increased use of spirometry over these 5 years may be because of aggressive spirometry training initiatives taken up by National societies such as the Indian Chest Society, Chest Research Foundation (CRF) and educational programmes carried out in various medical colleges. The Indian Chest Society has conducted ~40 programmes on spirometry across India, whereas CRF has trained >10,000 doctors in spirometry across the country. For a vast country such as India with over 1.5 million registered practitioners, this can only be a small step in the right direction.

We do not know whether the doctors who participated in our study had undergone any of the spirometry training programmes because the doctors were randomly selected. However, we agree that we missed capturing information regarding their participation in any of the training programmes. We accept this is an important omission.

Nonetheless, this impressive increase in the use of spirometry assures us that it is pragmatic to expect improvements in the use of spirometry in clinical practice in India and that efforts taken to improve awareness about spirometry have the potential to change clinical practice.

### Interpretation of findings in relation to previously published work

To the best of our knowledge, there is no previously published work on the use of spirometry in India.

### Strength and limitations of this study

To the best of our knowledge, this is the first time that the use of spirometry across India has been studied and documented. This information provides us with important insights and future directions.

Our study has several limitations. The response rates of 41% in 2005 and 54% in 2013 are modest. It is likely that those doctors who do not use spirometry or are not well versed with spirometry may have chosen not to respond, in which case the proportions of doctors who use spirometry in our study may be a gross underestimate. However, it could also be possible that the proportions are overestimated. One more limitation of our study is about the sample representativeness. Although the database used had doctors from different parts of India, it still may not be a true representation of our country.

### Implications for future research, policy and practice

GPs are the primary or the first point of contact for patients at the early stage of the disease, and therefore it is important for GPs to make an early and accurate diagnosis of OAD. Buffels *et al.* reported that when screening for airflow obstruction was initiated with spirometry by GPs the number of patients diagnosed with OADs doubled.^15^ Our study shows that awareness of spirometry among GPs is low. Only 10% of the GPs used spirometry in 2005, and this number did not change much in 2013. The results of our study suggest that GPs as a specific group of doctors should be educated and motivated to use spirometry in their clinic. Appropriate steps need be taken to increase the use of spirometry among GPs, such as education about spirometry and making spirometers available at an affordable cost.

Compared with 2005, the improvement in the use of spirometry was highest among Ps (224%) and CPs (31%) in the year 2013. The large increase in spirometry use among Ps is because of a small baseline in 2005—i.e., 21%. Despite such an apparently marked improvement even in 2013, only 16% of Ps reportedly used spirometry. There are greater challenges in using spirometry in the paediatric age group because it is an effort-dependent test that requires a lot of cooperation, and children below the age of 5 years are unlikely to be able to perform spirometry.

Spirometry is currently the gold-standard diagnostic test for asthma and COPD, and the lack of use of spirometry often leads to a large proportion of patients with OAD being under-diagnosed. Spirometers are not as economical as the blood pressure apparatus/syphgomamometer or the electrocardiography machine, it needs special training and expertise to perform the test properly, and it requires a good knowledge base to interpret the results properly. These are the likely reasons for spirometry not being popular among primary care physicians in routine clinical practice. The common reasons for underuse of spirometry in India were high costs of the spirometer, unaffordability of the test by the patients and busy schedule of the doctor. The average cost of spirometry in India is around 300–500 Indian rupees (i.e., 5–8 USD). This would be more than a day’s salary for over half of the Indian population, and it is therefore an expensive test. In India, >70% of patients pay through their pockets for medical services, and health insurance companies do not reimburse for lung function tests. Although the costs of spirometers have come down over the years, they are still perceived as an expensive tool to use in the clinic. Apart from the actual cost of the spirometer, physicians may even perceive that the time spent on spirometry to achieve three acceptable and two repeatable tests could be better used to see more patients. An average physician sees around 20–30 patients every day in the outpatient clinic.^16^ Physicians may therefore perceive that the use of spirometer is not only expensive but also time-consuming.

In 2005, 54% of the physicians reported not using spirometry because of high costs, and in 2013 the proportion reduced to 28%, suggesting that the cost of the spirometer became less important as a reason for not using spirometry. However, what increased was the perception that doctors did not have time to perform spirometry, which increased from 9% in 2005 to 32% in 2013. It is likely that as more doctors start using spirometry they realise that spirometry is a time-consuming test. Spirometry as a service is offered by only some central labs unlike chest X-ray or electrocardiography centres. Some doctors have trained their nurses or attendants to perform spirometry and therefore save their time. With such a high burden of OAD in India, it would seem appropriate to have a dedicated workforce of respiratory therapists or respiratory managers who may perform this role.

Another reason for not having a spirometer in the clinic is the prejudice that spirometry is a difficult test to perform and a difficult test to interpret. This issue needs to be addressed by conducting more and more spirometry training programmes. Apart from doctors, other supporting staff such as nursing staff, respiratory therapists and technicians should be actively trained and education initiatives should be targeted to this group at regular intervals.

One-third of the doctors were not aware about which predicted equations they were using. As of now, there are no reliable predicted values for spirometry available for the Indian population. Although some studies have derived predicted equations, they are limited to specific regions of India. In the absence of reliable Indian predicted values, physicians use different equations derived from the western population to determine OAD severity. This uncertainty of the use of predicted values used to determine asthma and COPD severity may affect or alter the diagnosis and severity grading of the disease. It is therefore essential for physicians to use appropriate predicted equations with appropriate correction factors. There is clearly an urgent need to derive predicted values for spirometry for the Indian population.

This study provides us with a very useful insight about the use of spirometry in India, and although the numbers of doctors who use spirometry is small the proportions are increasing. More doctors need to be made aware about the use of spirometry, they should be educated on how to perform the test and interpret it and this should be combined with lowering the costs of spirometers, so that more and more doctors will use this important diagnostic tool in their practice.

### Conclusion

Spirometry is a poorly utilised tool in primary care clinical practice in India, although the numbers have increased over the years. General practitioners and Ps, in particular, were poor users of spirometry. Cost of the spirometry was the main reason citied earlier, although with increasing use the long time required to perform spirometry became the main reason for not using spirometry. Creating awareness among primary care physicians about the important role of spirometry in clinical practice, educating them about how to perform and interpret spirometry, making spirometers available at an affordable cost and creating trained and skilled spirometry technicians are solutions to improve the use of spirometry in India.

## Materials and methods

We conducted two nationwide surveys in the years 2005 and 2013 among four groups of doctors: CPs, internists or GenPs, GPs and Ps.

### Sample selection and sampling strategy

There are an estimated 1.5 million registered doctors in India, 0.7 million of whom are trained in modern medicine, whereas the remaining are trained in alternative forms of medicine. However, there is no good registry of doctors across the country that is available or accessible. The Medical Council of India has a registry, but it is incomplete and not accessible. We therefore had to rely on other sources.

Cipla is a leading pharmaceutical company in India; they have a database of doctors all over India, which is updated every year. Their database has the address, contact details and email IDs of doctors. These doctors are approached by the field personnel of Cipla. The database currently has over 30,000 doctors, whereas in 2005 the database comprised 7,000 doctors and in 2013 it comprised 11,300 doctors. This was therefore the most reliable source of database available for this kind of study.

**Figure 4:**
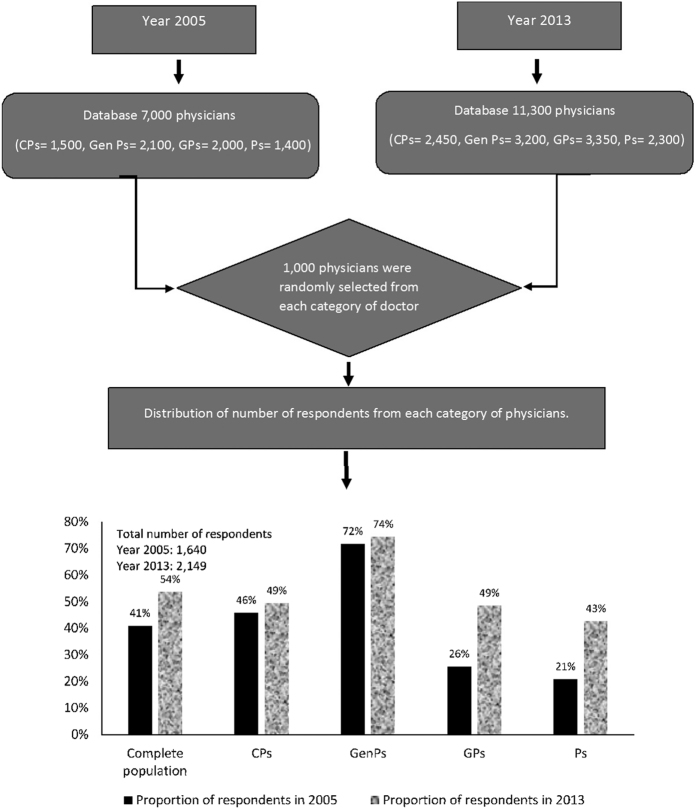
The study population and break-up between the years 2005 and 2013.

In 2005, we had a physician database of 7,000 physicians and in 2013 we had a database of 11,300 physicians, from which we randomly selected 1,000 physicians using SPSS command ‘random sample of cases’ from each group of CPs, GenPs, GPs and Ps (Figure 4) from across 52 cities and towns in 15 states of India.

A total of 4,000 physicians were therefore randomly selected in each survey. A sample size of 4,000 physicians was mainly based on logistical and operational feasibility. Moreover, we had no access to any other similar study conducted in India or otherwise to give us an estimate of the sample size that will give us an idea of sufficient power.

### Study tools

The research tool used was a one-page survey questionnaire designed and pilot-tested by us. The questionnaire was pilot-tested in 20 participants. The questionnaire was found to be well understood and did not lead to changes to the survey. The questionnaire included 15 questions that were divided into three sections: (1) physician demographic details, (2) practice and management of asthma and (3) use of spirometry.

Both the studies were jointly conducted by CRF, an academic research institute based in Pune, and Cipla, a generic multinational pharmaceutical company based in Mumbai, India. CRF designed the questionnaire, pilot-tested it, randomly selected the physicians from the database of doctors and performed data management and analysis, whereas Cipla contributed in the logistics with their field force personnel who visited the randomly selected physicians, briefed them about the study and handed over the documents containing information about study participation and the survey questionnaire, which the physicians were requested to fill. Cipla had no role in designing the survey, statistical analysis and write-up. The survey design, analysis and manuscript writing were solely done by authors from CRF.

Those physicians who consented completed the questionnaire themselves. The completed questionnaires were then sealed by the doctor in envelopes, which were sent back to CRF via courier. Data were entered using double data entry by two data entry operators. Our chief statistician checked the data for discrepancies. Discrepancies observed were then discussed with the data entry operators. Discrepant data were re-checked to see who made an error in transferring data. Corrections were then made once everybody agreed.

The same questionnaire and study methodology were used for the surveys in 2005 and 2013. The only change was an additional question in the 2013 survey regarding which predicted values the physicians used for spirometry tests.

### Statistical analysis

Data obtained in 2005 and 2013 surveys were analysed separately. Frequency and percentages of the items were measured for the four categories of practitioners. We also compared the differences in the mean percentage values between the two surveys using the *χ*^2^-test. All statistical analyses were performed using SPSS 11.5 Version (SPSS Inc., Chicago, IL,USA), and *P* values <0.05 were considered to be statistically significant.

## Figures and Tables

**Table 1:** Differences in reasons for not having a spirometer (2005 versus 2013)

*Reasons for not having a spirometer*	*Chest physician (%)*		P*-value*	*General physician (%)*		P*-value*	*General practitioner (%)*		P-*value*	*Paediatrician (%)*		P-*value*
	*2005*	*2013*		*2005*	*2013*		*2005*	*2013*		*2005*	*2013*	
Too expensive	35.37	28.89	0.317	60.51	28.22	<0.0001	53.85	34.79	<0.0001	48.50	19.33	<0.0001
Patients cannot afford the cost	41.46	31.11	0.121	44.87	31.63	<0.0001	57.95	31.63	<0.0001	25.75	18.33	0.058
Spirometry is not useful	8.54	1.48	0.011	4.36	3.41	0.459	4.62	2.92	0.286	6.59	4.67	0.378
Spirometry is difficult to perform	7.32	9.63	0.562	13.08	8.90	0.042	9.74	6.81	0.207	23.95	19.00	0.207
Busy to perform spirometry in clinic	4.88	34.81	<0.0001	9.23	38.26	<0.0001	11.79	30.41	<0.0001	10.18	23.67	<0.0001
Difficult to interpret	1.22	1.48	0.872	17.95	5.49	<0.0001	33.85	12.17	<0.0001	10.78	8.00	0.313
Refer patient to a chest specialist	0.00	2.22	<0.0001	0.26	10.23	<0.0001	1.54	18.25	<0.0001	0.00	12.33	<0.0001

Reasons and differences between 2005 and 213 for not having a spirometer by different groups of physicians.
